# Enantioselective Desymmetrization by Chiral Bifunctional H‐Bonding Organocatalysts

**DOI:** 10.1002/chem.202502172

**Published:** 2025-10-07

**Authors:** Michal Urban, Jan Veselý

**Affiliations:** ^1^ Department of Organic Chemistry Charles University Hlavova 8 Prague 128 43 Czech Republic

**Keywords:** asymmetric catalysis, bifunctional catalysis, desymmetrization, enatioselective organocatalytic reaction, H‐bonding catalysis

## Abstract

Enantioselective desymmetrization has emerged as an effective method for generating highly enriched chiral molecules from achiral compounds. In recent years, organocatalysis has played an increasingly prominent role in this field, offering metal‐free and environmentally benign alternatives to traditional asymmetric catalysis. This review focuses on organocatalytic approaches in desymmetrization reactions that utilize bifunctional hydrogen activation of the substrate by a chiral organocatalyst. The most common types of chiral bifunctional organocatalysts include cinchona alkaloids and their derivatives, such as thioureas, sulfonamides, and squaramides. The following text provides an overview of the enantioselective desymmetrization methods that have been described to date for *meso*‐anhydrides, *meso*‐diols, amines, small heterocyclic rings, and various carbonyl compound derivatives.

## Graphical abstract

Enantioselective desymmetrization has emerged as a highly efficient and conceptually elegant strategy for the construction of chiral molecules from readily available prochiral substrates. This review focuses on recent developments in organocatalytic desymmetrization reactions, with particular emphasis on approaches involving bifunctional activation through hydrogen bonding.

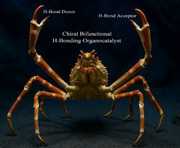

## 1 Introduction

Asymmetric synthesis plays a crucial role in organic chemistry, particularly in the construction of chiral molecules with high enantiomeric purity. Enantiomeric purity is essential for applications in the pharmaceutical, agrochemical, and materials industries.^[^ ^1^ ^]^ One effective strategy in enantioselective synthesis is desymmetrization.^[^ ^2^, ^3^ ^]^ In a desymmetrization reaction, a symmetrical or prochiral substrate is converted into an enantiomerically enriched product by selective activation of one of its enantiotopic or diastereotopic groups. This approach offers a high degree of atom economy and can rapidly increase molecular complexity from readily available starting molecules.^[^ ^4^, ^5^, ^6^, ^7^, ^8^ ^]^

**Scheme 1:**
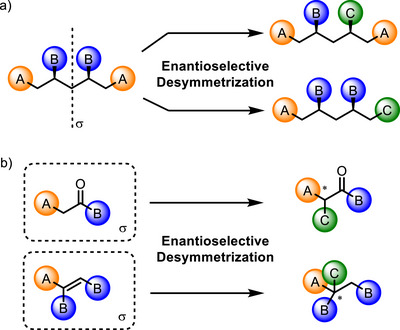
a) Enantioselective desymmetrization of *meso*‐compounds or achiral compounds. b) Enantiotopic facial differentiation.

Over the last 30 years, organocatalysis – a form of catalysis mediated by small organic molecules – has emerged as a highly effective method for enantioselective transformations, including desymmetrization reactions.^[^ ^9^, ^10^ ^]^ Among the various groups of chiral organocatalysts, chiral bifunctional hydrogen‐bond donors (H‐bonds), such as thioureas, urea, squaramides, and sulfonamides have gained significant prominence.^[^ ^11^, ^12^ ^]^ These chiral organocatalysts can activate substrates through a double noncovalent interaction. One functional group (usually a basic site, such as a tertiary amine or amidine) activates the nucleophile via a deprotonation or coordination bond, while the structural motif of the H‐bond donor simultaneously activates the electrophile via a hydrogen bond. These bifunctional catalysts excel in enantioselective transformations by providing well‐defined chiral and polarized reaction environments. Stereoselectivity is often controlled by both the fine balance of substrate orientation in the hydrogen bond‐activated chiral pocket and the differential stabilization of the diastereomeric transition states. The introduction of squaramide‐based catalysts has provided an advantage over earlier thiourea derivatives, due to their increased acidity and stronger hydrogen bonding, which in many cases can result in higher catalytic efficiency and stereoselectivity.^[^ ^13^ ^]^ Desymmetrization reactions catalyzed by bifunctional hydrogen‐interacting organocatalysts have been successfully applied to a wide range of classes of symmetric substrates, including *meso*‐anhydrides, *meso*‐epoxides, and *meso*‐aziridines, *meso*‐diols, prochiral ketones, diketones, dienones, and other 1,3‐dicarbonyl compounds. To date, several review articles have been written in this area, for example, by Spivey,^[^ ^14^ ^]^ Deng,^[^ ^15^ ^]^ and Bolm.^[^ ^16^ ^]^ After 2010, summary works in this field have been contributed by Villegas,^[^ ^17^ ^]^ Conon,^[^ ^18^ ^]^ and Dixon.^[^ ^3^ ^]^ However, an up‐to‐date overview of the current state‐of‐the‐art in this highly active field is lacking. In terms of definition, enantioselective desymmetrization is a reaction in which a *meso*‐compound or achiral compound is converted into a chiral substance by breaking symmetry. Most often, this involves breaking a suitable symmetry element, such as a plane (σ) or center (*i*) of symmetry or a rotational‐reflective axis (S*_n_*). It should be noted, however, that the desymmetrization reaction does not proceed at the point, where the symmetry element lies (Scheme 1a). This definition does not apply to reactions taking place at the point of symmetry, as is the case with enantioselective addition to carbonyl compounds or alkenes. Here, it is possible to distinguish between enantiomeric planes (Scheme 1b).

The aim of this review article is to provide a comprehensive overview of enantioselective organocatalytic desymmetrization reactions using chiral bifunctional H‐bonding organocatalysts. We pay special attention to enantioselective desymmetrizations of anhydrides with alcohols and thiols, and desymmetrization of small rings, such as epoxides, oxetanes, and aziridines. Later, we introduce the enantioselective preparation of various motifs using the desymmetrization of diols, diamines, and carbonyl compounds, including ketones or diketones, dienones or bis‐enones, cyclohexadienones, cyclopentadienones, and cyclohexadienones. Finally, we also focus on the synthetic applications of desymmetrization reactions in the total synthesis of natural compounds.

This review focuses on the use of organocatalysts structurally derived from ureas, thioureas, and squaramides as H‐bonding catalysts. Enantioselective organocatalytic desymmetrizations mediated by chiral phosphoric acids are outside the scope of this work. This topic has been thoroughly covered in review articles.^[^ ^3^, ^19^ ^]^

## 2 Enantioselective Desymmetrization of Anhydrides

### 2.1 Desymmetrization by Alcohols

**Figure 1:**
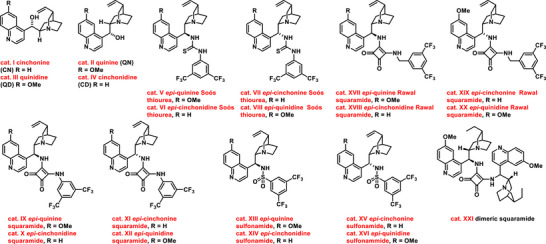
Chiral bifunctional organocatalysts based on cinchona alkaloids.

**Scheme 2:**
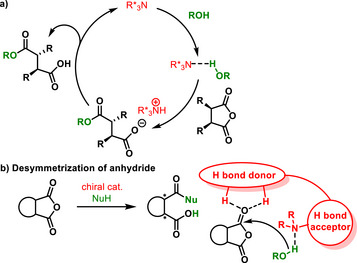
a) Catalytic cycle of desymmetrization of anhydrides. b) Activation modes of bifunctional H‐bonding catalyst with anhydride.

Chiral bifunctional organocatalysts are commonly utilized in enantioselective desymmetrization reactions. Classic examples of chiral organocatalysts, include cinchona alkaloid derivatives, which are typically found in the forms of thioureas, squaramides, and sulfonamides (Figure 1). One of the first functional groups used in organocatalytic enantioselective desymmetrizations is the anhydride moiety. The mechanistic understanding of organocatalytic enantioselective desymmetrization of anhydrides with alcohols remains a matter of debate; however, base catalysis, supported by kinetic data, is today widely regarded as the most plausible model (Scheme 2a). When cinchona alkaloids are employed as the basic part of the organocatalyst, the tertiary quinuclidine amine effectively promotes alcohol activation, enabling the following nucleophilic anhydride ring‐opening. It should be noted that, bifunctional activation of the substrate by the catalyst can be expected. The carbonyl group of the anhydride can be activated via a hydrogen bond of the quinoline alkaloid, thiourea, or squaramide (Scheme 2b).

**Scheme 3:**
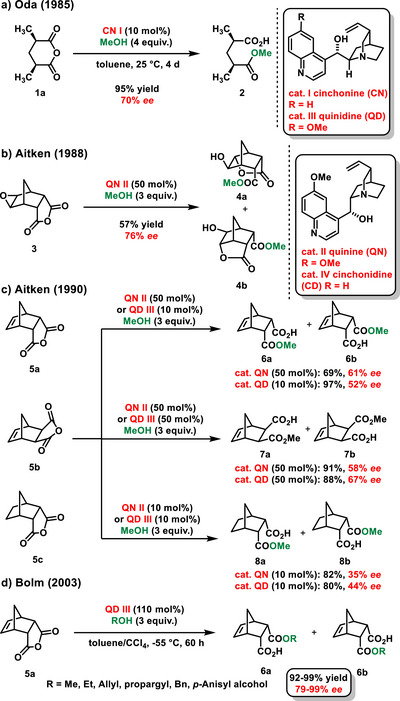
Enantioselective organocatalytic desymmetrization of *meso*‐anhydride with methanol.

The first chiral organocatalysts used for enantioselective desymmetrization of anhydrides were Cinchona alkaloids. In 1985 and 1987, Oda published the first asymmetric desymmetrization of *cis*‐dimethylglutaric anhydride **1** with methanol, using cinchonine **I** as the catalyst. This methanolytic reaction produced hemiesters **2** in high yield (95%) with an enantiomeric excess of 70% *ee* (Scheme 3a).^[^ ^20^ ^]^ Aitken published a similar work soon after, focusing on the organocatalytic desymmetrization of *meso*‐epoxy anhydride **3** using methanol to produce lactones **4a,b**. The reaction catalyzed by quinine **II** resulted in a yield of 57% with an enantiomeric excess of 76% *ee* (Scheme 3b).^[^ ^21^ ^]^ Two years later, Aitken published an enantioselective desymmetrization of bridged tricyclic anhydrides **5a,b,c**, using quinoline alkaloids **II** and **III** as catalysts (Scheme 3c).^[^ ^22^ ^]^ In 2000, Bolm built upon this research by publishing an asymmetric desymmetrization method for various tricyclic and bicyclic *meso*‐anhydrides **5** using methanol.^[^ ^23^ ^]^ Three years later, this work was expanded to include the use of various alcohols in the desymmetrization of *meso*‐tricyclic anhydrides **5a** (Scheme 3d).^[^ ^24^ ^]^

**Scheme 4:**
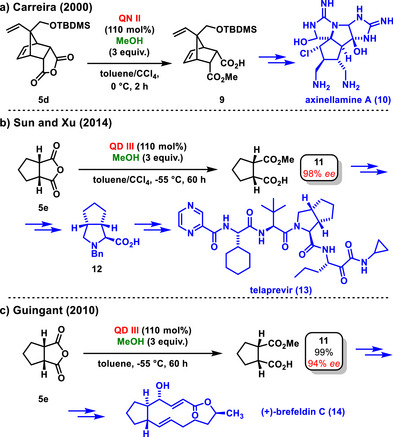
Application of enantioselective organocatalytic desymmetrization of *meso*‐anhydrides with methanol in total synthesis biological active compounds.

Bolm's concept of organocatalytic enantioselective desymmetrization of *meso*‐tricyclic anhydride **5d** was utilized by Carreira in the total synthesis of axinellamine A (**10**). A crucial step in the synthesis produced the hemiester **9** in quantitative yield with an enantiomeric excess of 93% *ee* (Scheme 4a).^[^ ^25^ ^]^ Similarly, Bolm's work has also been applied in the synthesis of telaprevir (**13**), a protease inhibitor of the hepatitis C virus.

Authors Sun and Xu, used asymmetric desymmetrization of a bicyclic *meso*‐anhydride **5e** using methanol and catalyzed by quinidine **III** in stoichiometric amounts. The corresponding hemiester **11** was obtained with 98% *ee*, and used in a subsequent six‐step synthesis of a key telaprenavir intermediate, pyrrolidine derivative **12** (Scheme 4b).^[^ ^26^ ^]^ Notably, the initial step in the synthesis of antibiotic (+)‐brefeldin C (**14**) and its analogues involved an enantioselective desymmetrization of the *meso*‐anhydride **5e** by methanol. Hemiester **11** was obtained in a high yield (99%) and with enantiomeric excess (94% *ee*) (Scheme 4c).^[^ ^27^ ^]^

**Scheme 5:**
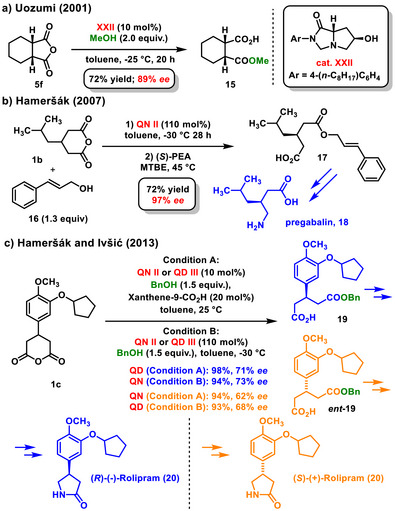
a) Enantioselective organocatalytic desymmetrization of *meso*‐bicyclic anhydride with methanol; b) and c) Alcoholysis of glutaric anhydrides the synthesis of pregabalin and enantiomers of rolipram.

In 2001, Uozumi published an enantioselective desymmetrization of *meso*‐cyclic anhydrides **5f** with methanol, marking the first use of a different type of chiral organocatalyst, rather than the quinoline alkaloids. A derivative of hexahydro‐1H‐pyrroloimidazole **XXII** effectively catalyzed this desymmetrization, yielding the monoester **15** at 72% with an enantiomeric excess of 89% *ee* (Scheme 5a).^[^ ^28^ ^]^ In 2007, Hameršák published a method for synthesizing pregabalin (**18**). The key step of the synthesis was the enantioselective desymmetrization of a derivative of glutaric anhydride **1b** using cinnamyl alcohol **16**. The reaction was successfully catalyzed by quinine **II**, resulting in the desired hemiester **17** with a yield of 72%, and an enantiomeric excess of 72% *ee*. The enantiomeric excess of the product was further improved to 97% *ee* through recrystallization with (*S*)‐phenylethylamine (Scheme 5b).^[^ ^29^, ^30^ ^]^A few years later, Hameršák and Ivšić utilized asymmetric desymmetrization of anhydrides **1c** in the total synthesis of both enantiomers of rolipram (**20**) (Scheme 5c).^[^ ^31^ ^]^ The enantioselective desymmetrization was catalyzed with quinine **II** or quinidine **III** in both catalytic and stoichiometric amounts. When using a catalytic amount of the cinchona alkaloid (**II**, **III**), xanthen‐9‐carboxylic acid was added to reverse the enantiocontrol of the process. The enantioselective alcoholysis of anhydride **1c** produced hemiesters **19** and ***ent*‐19** in good yields (93–98%) and with enantiomeric excesses ranging from 62% to 73% *ee*.

**Scheme 6:**
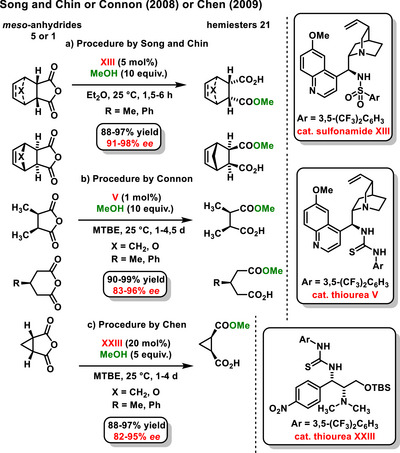
Enantioselective organocatalytic desymmetrization of *meso*‐anhydride with methanol, catalyzed by thiourea or sulfonamide described by Song and Chin a), Connon b), and Chen c).

**Figure 2:**
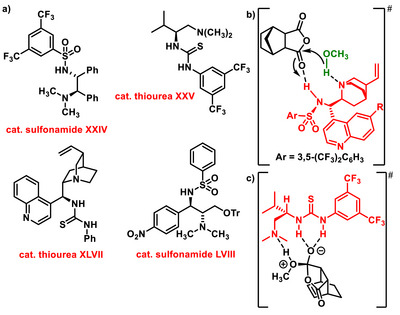
a) Example chiral bifunctional sulfonamides and thioureas. b) Transition‐state structure proposed by Hofmeister and Kohen. c) Transition‐state structure proposed by Pedrosa.

The first example of desymmetrizing anhydrides using chiral bifunctional sulfonamides **XIII**, derived from chinchona alkaloid was reported by Song and Chin in 2008. They conducted organocatalytic methanolysis of anhydrides **5** or **1**, successfully producing hemiesters **21** with good yields (88–97%) and high enantiomeric purity (91–98% *ee*) (Scheme 6a).^[^ ^32^ ^]^ In the following study, the same authors conducted a desymmetrization of anhydrides using a chiral thiourea derived from *epi*‐quinine **V**. They noted an intriguing influence of temperature and solvent concentration on the reaction's enantioselectivity.^[^ ^33^ ^]^ In 2009, Song expanded this study to a polymer‐supported chiral sulfonamide **XIII** derived from *epi*‐quinine, which catalyzed the enantioselective desymmetrization of *meso*‐anhydrides **5** or **1** with methanol. Hemiesters **21** were obtained in quantitative yields with enantiomeric excesses ranging from 89 to 97%.^[^ ^34^ ^]^ In 2017, building on the work of Song and Chin, an extensive theoretical study on the mechanistic aspects of organocatalytic enanthioselective desymmetrization of cyclic *meso*‐anhydrides using alcohols was reported by Hofmeister and Kohen (Figure 2b).^[^ ^35^ ^]^

In 2008, Connon introduced the concept of enantioselective desymmetrization of simple, bicyclic, and tricyclic *meso*‐anhydrides **5** using methanol under catalysis with chiral thiourea derived from cinchona alkaloids **V**. Only a tiny amount of catalyst (1 mol%) was sufficient for successful enantioselective desymmetrization, affording the corresponding monoesters in high yields (90–99%) and with excellent enantiomeric excesses (83–96% *ee*) (Scheme 6b).^[^ ^36^ ^]^ Subsequently, Chen reported a similar study on the enantioselective desymmetrization of *meso*‐anhydrides **5** or **1,** with methanol under catalysis with a new type of chiral thiourea **XXIII** (Scheme 6c).^[^ ^37^ ^]^

Not only methanol, but also other alcohols have been used for the enantioselective desymmetrization of *meso*‐anhydrides. For example, Sano utilized benzyl alcohol as a nucleophile for the desymmetrization of anhydride **5** or **1**. The reaction was successfully catalyzed by a chiral bifunctional sulfonamide derived from (*R*,*R*)‐1,2‐diphenylethylene‐1,2‐diamine **XXIV** (Figure 2). The obtained hemiesters were then, in‐situ converted to the corresponding diesters using trimethylsilyldiazomethane in 80–99% yield and enantiomeric excess in the range of 83–98% *ee*.^[^ ^38^ ^]^ In 2010, Pedrosa utilized chiral thioureas derived from L‐valine **XXV** (Figure 2a) as bifunctional catalysts for the enantioselective desymmetrization of various *meso*‐anhydride derivatives **5** or **1**. Alongside methanol, he also used allyl and benzyl alcohol; however, *tert*‐butanol was found to be unreactive in this transformation. The resulting monoester derivatives were obtained in good yields, ranging from 55% to 99%, and with high enantiomeric purity, up to 98% *ee*.^[^ ^39^ ^]^ Based on his calculations, Pedrosa postulated a transition state structure for the desymmetrization of anhydride using chiral thiourea (Figure 2c). Prior to Pedrosa's work, Song had published study on the asymmetric desymmetrization of *meso*‐glutaric anhydrides **1** using different alcohols. He successfully catalyzed the reaction with a previously validated chiral sulfonamide derived from quinine **XIII**, producing hemiesters with yields between 81% and 98%, and with enantiomeric excesses ranging from 90% to 98% *ee*.^[^ ^40^ ^]^

**Scheme 7:**
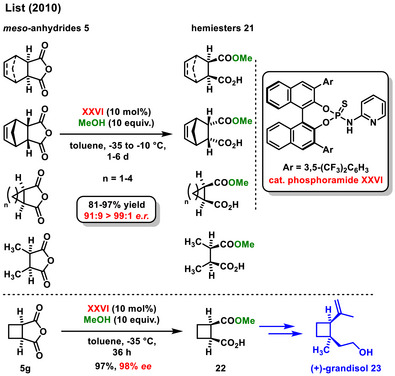
Enantioselective desymmetrization of *meso*‐anhydride with alcohol catalyzed by derivative of thiophosphoramide.

In that time, Aviyente published a theoretical study on the mechanism of enantioselective desymmetrization of cyclic *meso*‐anhydrides using alcohols. In this study, she employed amino alcohols as chiral organocatalysts. Utilizing DFT quantum mechanics, she supported the mechanism based on the deprotonation of the alcohol by the tertiary amine in the catalyst structure, resulting in the formation of a hydrogen bond. This process is followed by an attack on the carbonyl carbon of the *meso*‐anhydride, resulting in ring opening. Additionally, a second proposed mechanism involves the nucleophilic addition of the tertiary amine of the catalyst to the carbonyl carbon of the anhydride, but this approach requires significantly higher activation energy.^[^ ^41^, ^42^ ^]^ At the beginning of 2010, the List group developed a new type of bifunctional organocatalyst derived from a BINOL structure, thiophosphoramide derivative **XXVI** bearing a pyridine moiety. The catalyst **XXVI** was utilized in the asymmetric methanolysis of various *meso*‐anhydride derivatives **5**. The effectiveness of this approach was later demonstrated in the total synthesis of (+)‐grandisol (**23**). The key hemiester **22,** was obtained with good yield (97%) and high enantiomeric excess (98% *ee*) (Scheme 7).^[^ ^43^ ^]^

**Scheme 8:**
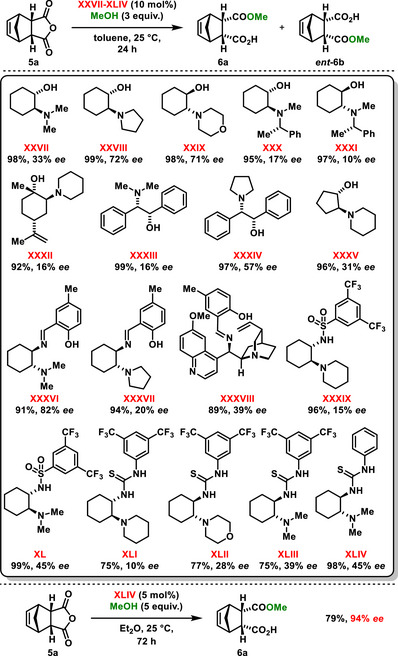
The use of different organocatalysts in the enantioselective desymmetrization of anhydrides by methanol, according to Bolm.

Another example of the application of organocatalytic enantioselective desymmetrization was presented by Chen in the total synthesis of (+)‐biotin.^[^ ^44^ ^]^ A comprehensive study of asymmetric desymmetrization of anhydrides, using alcoholysis was conducted by the Bolm's group (Scheme 8).^[^ ^45^ ^]^ They tested a wide variety of chiral bifunctional organocatalysts, including derivatives of amino alcohols **XXVII–XXXVIII**, aminosulfonamides **XXXIX**, **XL**, and bifunctional thioureas **XLI‐XLIV**. After identifying the most effective catalyst in terms of yield and reaction selectivity, the corresponding thiourea **XLIV,** was evaluated in enantioselective desymmetrization methanolysis, involving various *meso*‐anhydrides **5**, affording hemiesters **6** in high yields with high enantiomeric purity.

Another example of using asymmetric desymmetrization of anhydrides is found in the enantioselective synthesis of 4‐aryl‐substituted 5‐carboxy‐3,4‐dihydropyridin‐2‐one derivatives. This work was published by Huang,^[^ ^46^ ^]^ who employed a chiral thiourea **V** derived from quinine as the organocatalyst (2 mol%) for the enantioselective reaction. The study involved extensive testing of various solvents in the reaction between the anhydride derivative and 10 equivalents of methanol. Ultimately, 2‐methyltetrahydrofuran was identified as the optimal solvent, providing the best selectivity (80% *ee*) for the reaction.

In 2011, Song continued in his research by testing dimeric squaramides **XXI** derived from quinoline alkaloids in the enantioselective methanolysis of *meso*‐glutaric anhydride derivatives. The process produced the targeted hemiester in high yields, ranging from 91% to 98%, with improved enantioselectivity (89–93% *ee*).^[^ ^47^ ^]^ Interestingly, a the dimeric squaramide organocatalyst exhibited a stable enantiomeric excess in the desymmetrization reaction, across various concentrations (c = 0.025–0.20 M), contrary monomeric squaramides and thioureas, which were efficient only at a high dilution of the reaction mixtures (c = 0.025 M). Also, Bolm's work involved the preparation of non‐alkaloid chiral sulfoximine‐based thioureas, which were utilized in the asymmetric desymmetrization of *meso*‐anhydrides using methanol. Unfortunately, this catalyst produced racemic mixtures of hemiesters with a yield of 66%.^[^ ^48^ ^]^

**Scheme 9:**
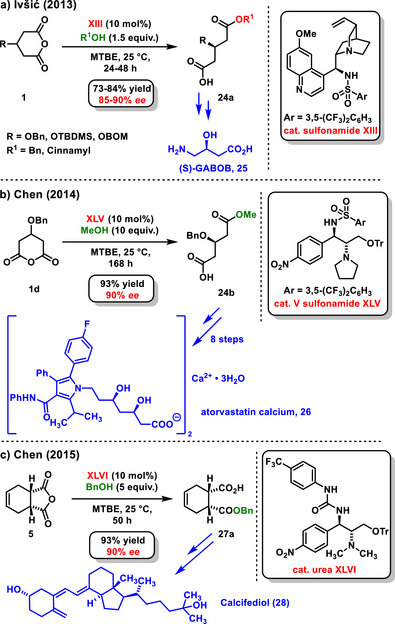
Application of enantioslective desymmetrization of anhydrides in total synthesis of biologically active compounds and drugs.

Soon after, in 2013, Chen reported an asymmetric desymmetrization of cyclic *meso*‐anhydride **5** or **1** using bifunctional sulfonamide **XLV**. In addition to classical methanolysis, he tested various alcohols in the process. High yields of the corresponding hemiesters were obtained, ranging from 83% to 97%, along with enantiomeric excesses between 17% and 93% *ee*. The developed method was successfully applied to prepare a key intermediate in the synthesis of P2X7 receptor antagonist.^[^ ^49^ ^]^ Nearly simultaneously, Ivšić published another study on enantioselective desymmetrization under catalysis with chiral bifunctional sulfonamide derived from quinine **XIII**, affording intermediate **24a** for the preparation of (*S*)‐γ‐amino‐β‐hydroxybutyric acid (**25**) ((*S*)‐GABOB, Scheme 9a).^[^ ^50^ ^]^

A year later, Chen published a work on the asymmetric synthesis of atorvastatin calcium (**26**) (Lipitor). In this study, he employed organocatalytic enantioselective desymmetrization of a cyclic anhydride to prepare hemiester **24b**, achieving a yield of 93% as an intermediate for the subsequent eight‐step total synthesis. The chiral bifunctional sulfonamide **XLV** effectively catalyzed the enantioselective reaction, resulting in high enantioselectivity (90% *ee*) (Scheme 9b).^[^ ^51^ ^]^ The same group also utilized chiral bifunctional urea **XLVI** to achieve the enantioselective desymmetrization of bicyclic *meso*‐anhydride **5** using benzyl alcohol. During the optimization of reaction conditions, it was observed that diluting the reaction mixture had a positive impact on the enantioselectivity. The resulting hemiester **27a** was obtained with a high yield of 93% and an enantiomeric excess of 90% *ee*. After subsequent recrystallization, the enantiomeric purity was improved to 96% *ee*. The authors then employed the prepared hemiester **27a** to synthesize a precursor for Calcifediol, a Vitamin D_3_ analogue (Scheme 9c).^[^ ^52^ ^]^ Four years later, Peng and Chen published a comprehensive paper on the enantioselective desymmetrization of various derivatives of succinic and glutaric anhydrides, as well as different alcohols. In this study, a wide range of bifunctional chiral organocatalysts, based on chloramphenicol were synthetized, which were then utilized in the asymmetric alcoholysis of the aforementioned anhydrides.^[^ ^53^ ^]^

**Scheme 10:**
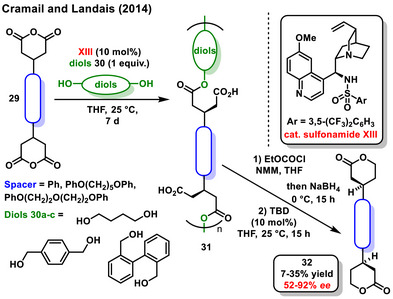
Application of enantioselective organocatalytic desymmetrization of *bis*‐cyclic anhydrides in the synthesis of chiral polyesters.

An interesting application of enantioselective organocatalytic desymmetrization of cyclic anhydrides **29** is the synthesis of chiral polyesters **31**, as reported by Cramail and Landais.^[^ ^54^ ^]^ In their work, *bis*‐cyclic anhydrides **29** were polymerized with various aliphatic and benzylic diols **30a‐c**, and a sulfonamide derived from *epi*‐quinine **XIII** was employed as a chiral organocatalyst. The developed process produced both linear polyesters, with a maximum degree of polymerization (n_max_) of 14, and cyclic polyesters, with n_max_ of 7. To determine the enantiomeric excess, the resulting polymers were degraded to their corresponding bis‐lactones **32** (Scheme 10).

In 2014, Chen reported two works on the use of nitroallyl alcohols for enantioselective desymmetrization of anhydrides.^[^ ^55^, ^56^ ^]^ In both studies, a chiral thiourea derived from *epi*‐cinchonidine **XLVII** was employed as a catalyst for the asymmetric induction. In one study, the enantioselective desymmetrization of glutaric anhydride **1** derivatives was conducted, while the other focused on succinic anhydrides **5**. The formation of corresponding hemiesters proceeded with good yields and high enantiocontrol in both studies. Notably, in the desymmetrization of the glutaric anhydride derivative, racemic nitroallyl alcohol was used, leading to the observation of kinetic resolution of the nitroallyl alcohol.

In 2017, Sierra and de la Torre investigated the organocatalytic desymmetrization of *meso*‐ferrocene anhydrides using ethanol. They found that a dimeric squaramide derived from chinchona alkaloid **XXI** was the most effective organocatalyst in terms of yield and selectivity for the reaction, producing the final hemiesters with an isolated yield of 60% and an enantiomeric excess of 98% *ee*.^[^ ^57^ ^]^

At this point, it is appropriate to discuss the possible limitations of enantioselective desymmetrization of anhydrides. The main limitation arises from the substrate scope dictated by symmetry requirements. For desymmetrization to generate a new stereocenter, the starting anhydride must possess either C_2_‐symmetry or be prochiral. Steric effects can further restrict applicability, as bulky anhydrides may hinder catalyst access, thereby diminishing both reactivity and enantioselectivity. Nevertheless, bifunctional sulfonamide catalysts derived from cinchona alkaloids have frequently proven effective in the enantioselective organocatalytic desymmetrization of anhydrides, often delivering high performance even at low catalyst loadings. Importantly, these are not the only viable systems: a broad range of chiral bifunctional H‐bonding organocatalysts, particularly those based on thioureas and squaramides, has also been explored with success.

**Scheme 11:**
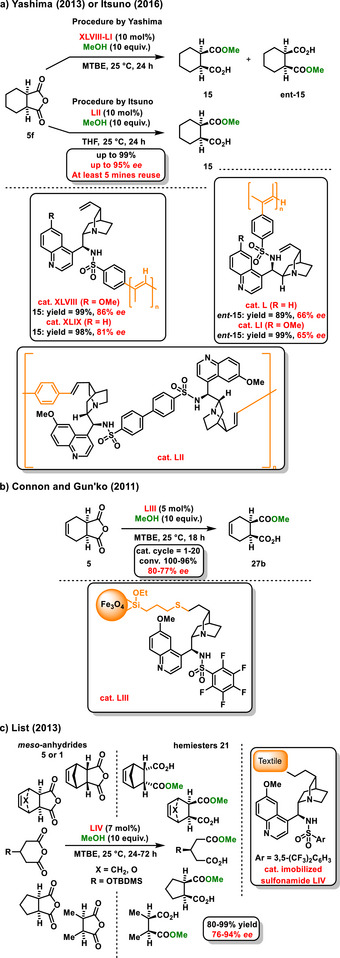
Examples of immobilized chiral organocatalysts applied in an enantioselective desymmetrization reaction.

In addition to homogeneous catalytic systems, chiral bifunctional organocatalysts have also been immobilized on polymer chains to improve their practical applicability. In 2013, Yashima introduced the use of polymer‐supported chiral organocatalysts for the enantioselective desymmetrization of succinic‐type of anhydrides.^[^ ^58^ ^]^ He designed and synthesized innovative helical poly(phenylacetylenes) containing chiral amides and sulfonamides derived from cinchona alkaloids **XLVIII‐LI**. The enantioselective methanolysis of the anhydride derivative **5f** using chiral polymeric organocatalysts provided hemiesters **15** or ***ent*‐15** with excellent yields and good enantiomeric excesses (Scheme 11a). However, when comparing the enantiomeric excesses of the hemiesters generated by polymeric and monomeric catalysts (84–97% *ee*), it was observed that the polymeric organocatalysts operate with lower enantiocontrol (65–86% *ee*). Three years later, Itsuno developed a series of polymeric chiral sulfonamides derived from quinoline alkaloids using Mizoroki‐Heck polymerization. He utilized these new polymeric organocatalysts **LII** for the enantioselective desymmetrization of a bicyclic anhydride **5f** with methanol. After five iterations of the reaction, the optically active hemiester **15** was isolated with a yield of 99% and an enantiomeric excess of 95% (Scheme 9a).^[^ ^59^ ^]^

A fascinating example of an immobilized chiral organocatalyst is its integration with magnetic nanoparticles. This approach was developed by Connon and Gun'ko, who attached a chiral sulfonamide to magnetic nanoparticles **LIII**. They utilized this setup in the catalytic enantioselective desymmetrization of succinic anhydride **5** with methanol. After the first catalytic cycle, the corresponding hemiester **27b** was produced with an enantiomeric excess of 80%. Interestingly, after twenty catalytic cycles, it achieved an enantiomeric excess of 77% *ee* (Scheme 11b).^[^ ^60^ ^]^ Another interesting example was disclosed by List in 2013, who described immobilizing a chiral sulfonamide derived from quinine onto nylon textile using ultraviolet light. The catalyst **LIV** demonstrated good stability, activity, and recyclability in the enantioselective desymmetrization of *meso*‐anhydrides with methanol. Additionally, a range of different anhydrides **5** or **1** were tested for their applications. The resulting hemiesters **21** were isolated in good yields and exhibited excellent enantiomeric excesses (Scheme 11c).^[^ ^61^ ^]^

**Scheme 12:**
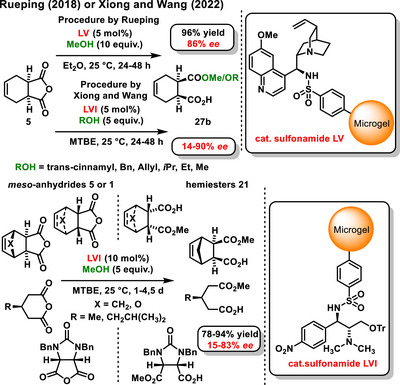
A highly stereoselective and recyclable microgel‐supported bifunctional sulfonamide organocatalyst for asymmetric alcoholysis of *meso*‐cyclic anhydrides.

In 2018, Rueping reported the work on the development of chiral sulfonamide **LV** based on a temperature‐responsive polymer microgel. The developed catalyst was utilized in the enantioselective desymmetrization of a cyclic *meso*‐anhydride derivative **5** using methanol. The reaction produced the hemiester **27b** in good yield (96%) and with a high enantiomeric excess (86% *ee*), even after ten subsequent catalytic cycles (Scheme 10).^[^ ^62^ ^]^ Building upon this work, Xiong and Wang further developed the concept by linking a different type of bifunctional sulfonamide to the microgel. Selected chiral microgel‐supported organocatalyst **LVI** was then applied to catalyze the asymmetric desymmetrization of cyclic *meso*‐anhydrides derivatives **5** or **1** using various alcohols (Scheme 12).^[^ ^63^ ^]^

**Scheme 13:**
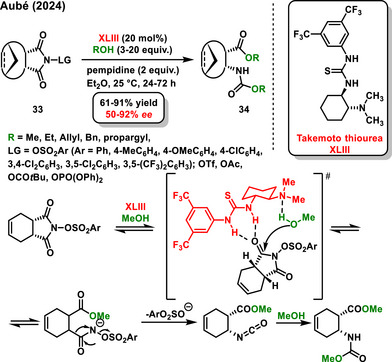
Synthesis of cyclic β‑amino acid derivatives by desymmetrization and Lossen rearrangement of *N*‑sulfoxy *meso*‐succinimides.

Recently, Aubé reported a study, that expands on the concept of organocatalytic desymmetrization of anhydrides using alcohol. In this concept, *N*‐sulfoxy *meso*‐succinimide **33,** served as a substrate in the desymmetrization reaction, facilitated by Lossen's rearrangement. The reaction was effectively catalyzed by the chiral bifunctional Takemoto thiourea **XLIII**, resulting in the formation of the target chiral cyclic β‐amino acid derivatives **34**. The yield of the asymmetric reaction ranged from 61% to 91%, with an enantiomeric excess between 50% and 92% *ee* (Scheme 13).^[^ ^64^ ^]^ The proposed mechanism employs the attack of activated methanol on the *Re*‐face of the *N*‐sulfoxy *meso*‐succinimide derivative. Subsequently, an irreversible Lossen rearrangement of the *O*‐sulfonyl hydroxamate ester occurs, giving isocyanate, which reacts with another equivalent of methanol to form the target product.

### 2.2 Desymmetrization of Anhydrides by Thiols

**Scheme 14:**
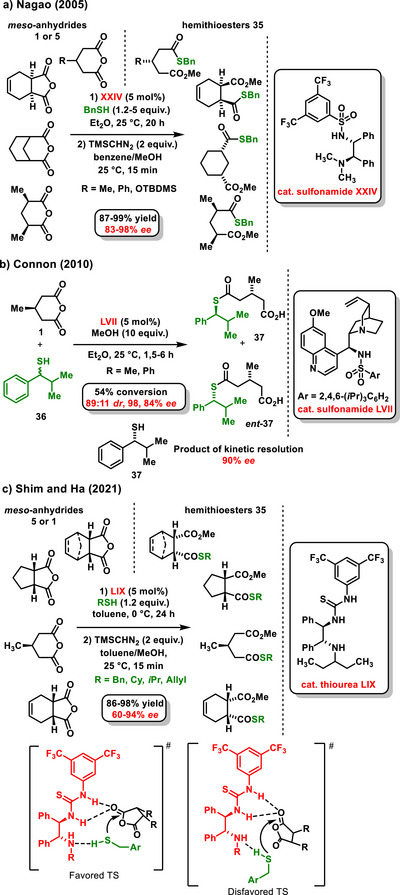
Enantioselective catalytic thiolysis of achiral cyclic anhydrides utilizing a bifunctional chiral sulfonamide or thiourea.

Similar to alcohols, thiols have been utilized in the enantioselective desymmetrization of anhydrides. One of the earliest studies in this area, reported by Nagao in 2005, focused on the asymmetric organocatalytic thiolysis of prochiral cyclic anhydrides **5** or **1** using benzylthiol (Scheme 14a).^[^ ^65^ ^]^ In this work, a chiral sulfonamide **XXIV** was used as an organocatalyst to produce monothioesters **33** in good yields (87–99%) and with high enantiomeric purity (83–98% *ee*). Nagao's work was followed by Connon, who published one example of enantioselective desymmetrization of a glutaric anhydride derivative **1** by cyclohexanethiol using a chiral bifunctional thiourea derived from *epi*‐quinine **V** as a catalyst.^[^ ^66^ ^]^ Two years later, the same group reported an extensive study where variously substituted racemic secondary benzylthiols **35** were tested in the asymmetric desymmetrization of a 3‐substituted glutaric anhydride derivative **1**. The reaction was catalyzed by a chiral bifunctional sulfonamide derived from *epi*‐quinine **LVII**, and kinetic resolution of the corresponding thiols occurred during the reaction. Thioesters **36** or ***ent*‐36** were prepared with good diastereoselectivity (89:11 *dr*) and high enantiomeric excesses (98% and 84% *ee*) (Scheme 14b).^[^ ^67^ ^]^

In 2013, Chen and his group disclosed an extensive study focusing on the desymmetric thiolysis of cyclic succinic **5** and glutaric anhydrides **1**. Considering the selectivity and yield of the reaction, bifunctional sulfonamide **LVIII** (Figure 2) emerged as the most suitable chiral organocatalyst. All the prepared hemithioester derivatives using different thiols were obtained at high yields (78–94%) with high enantioselectivity (81–92% *ee*). The synthetic utility of asymmetric desymmetrization using thiols was subsequently demonstrated in the total synthesis of pregabalin.^[^ ^68^ ^]^ A recent example of organocatalytic enantioselective desymmetrization of cyclic anhydrides **1** or **5** using thiols is the work by Shim and Ha. The authors successfully conducted thiolysis of anhydrides **1** or **5** with a chiral diamine thiourea **LIX**, yielding hemithioester derivatives **33** in good yields 86–98% with an enantiomeric purity in the range 60% and 94% *ee* (Scheme 14c).^[^ ^69^ ^]^ The authors also proposed possible transition states involving bifunctional activation of starting materials through hydrogen bonds. In this context, they discussed the role of alkyl substituents in the *meso*‐anhydride framework, which must be oriented to avoid steric hindrance to the nucleophilic attack of thiol.

## 3 Desymmetrization of Small Rings Containing Heteroatom

**Scheme 15:**
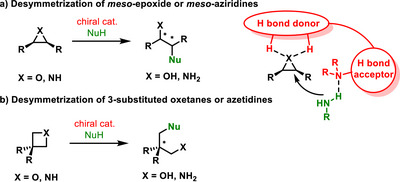
General scheme of desymmetrization of small rings.

Examples of small rings that undergo organocatalytic enantioselective desymmetrization include epoxides, oxetanes, and aziridines. We will begin by focusing on epoxides and their organocatalytic enantioselective desymmetrizations, which typically involve the opening of a polarized and strained ring (Scheme 15). Comprehensive reviews of this field were provided by Wang^[^ ^70^ ^]^ and Lattazi^[^ ^71^ ^]^ nearly ten years ago.

**Scheme 16:**
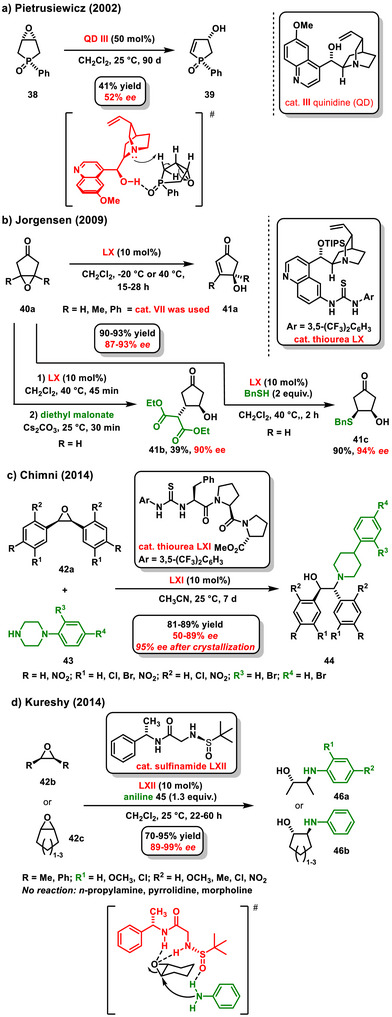
a) Enantioselective desymmetrization of a phospholene *meso*‐epoxide catalyzed by Quinidine. b) Organocatalytic desymmetrization of *meso*‐epoxycyclopentanone by Jørgensen. c) and d) Enantioselective desymmetrization of a *meso*‐epoxide with amines catalyzed by chiral thiourea or sulfinamide.

The first organocatalytic example of desymmetrization of chiral epoxide involves the use of quinoline alkaloids. In 2002, the Pietrusiewicz group reported the work on the enantioselective desymmetrization of a chiral phospholene *meso*‐epoxide **38** catalyzed by quinidine **III**. The reaction is classified as a β‐elimination, which facilitates the formation of a cyclic allylic alcohol **39** that also possesses a phosphorus stereogenic center. The final product **39** was obtained with a yield of 41% and an enantiomeric excess of 52% *ee* (Scheme 16a).^[^ ^72^ ^]^ In the proposed transition state, the activation of phosphorylated oxygen via the hydrogen bond of the OH group is absolutely essential. Furthermore, it can be seen that the tertiary amine deprotonates the hydrogen atom, which is in the *anti*‐position relative to the epoxide oxygen.

In 2009, Jørgensen utilized chiral thioureas derived from quinoline alkaloids **LX**, **VIII** to synthesize 4‐hydroxycyclopent‐2‐enone derivatives **41** from *meso*‐epoxycyclopentanones **40** through a β‐elimination process. The resulting cyclic allylic alcohols **41a** were obtained at high yields (90–93%) and with high enantiomeric excess (87–93% *ee*) (Scheme 16b).^[^ ^73^ ^]^ Additionally, it has been demonstrated that the process can be expanded to *one‐pot* desymmetrization/Michael addition sequence, producing highly substituted 4‐hydroxycyclopentanones **41b**, **41c**.

The study conducted by Chimni explored the use of bifunctional chiral thioureas containing a peptide chain **LXI** in the enantioselective desymmetrization of *meso*‐epoxides **42a** (Scheme 12c).^[^ ^74^ ^]^ In this process, *N*‐phenylpiperazine derivatives **43** served as the nucleophiles for the opening of the epoxide ring. In addition to *N*‐phenylpiperazine derivatives, aniline derivatives **45** have also been utilized for enantioselective desymmetrization of *meso*‐epoxides **42b** or **42c**. A chiral sulfinamide **LXII** was used to catalyze the process, affording the products **46a** or **46b** in good yield and selectivity in the study conducted by Kuresha's group (Scheme 16d).^[^ ^75^ ^]^ The synthesized chiral β‐amino alcohols **46a** or **46b** were isolated in yields ranging from 70% to 95% along with high enantiomeric excesses (89–99% *ee*). It is important to note that aliphatic primary amines or cyclic secondary amines (pyrrolidine, morpholine) did not provide the desired amino alcohols in this reaction.

**Scheme 17:**
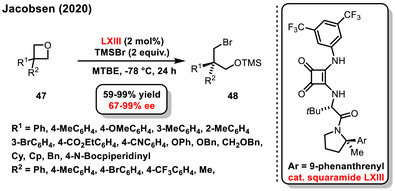
Enantioselective desymmetrization of a 3,3‐disubstituted oxetanes catalyzed by bifunctional chiral squaramide.

Another type of substrate that undergoes enantioselective desymmetrization is the 3‐substituted oxetane motif. In 2020, Jacobsen published research based on the enantioselective opening of substituted oxetane derivatives **47** (Scheme 17).^[^ ^76^ ^]^ This process involves the addition reaction of trimethylsilyl bromide to the oxetane motif, producing protected 1,3‐bromohydrin derivatives **48** in good yields (59–99%) and selectivity (67–99% *ee*). A chiral bifunctional squaramide derivative **LXIII** was utilized as the organocatalyst for this reaction.

**Scheme 18:**
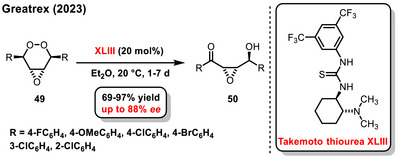
Enantioselective desymmetrization of a *meso*‐endoperoxide catalyzed by chiral Takemoto's thiourea.

A notable work from recent years was the enantioselective desymmetrization of cyclic *meso*‐endoperoxides **49**, developed by Greatex using the Kornblum‐DeLaMare rearrangement. The rearrangement promoted by a chiral bifunctional, Takemoto's thiourea **XLIII,** provided *γ*‐hydroxyketones **50** in both good yields (69–97%) and enantiomeric purity (14–88% *ee*) (Scheme 18).^[^ ^77^ ^]^

**Scheme 19:**
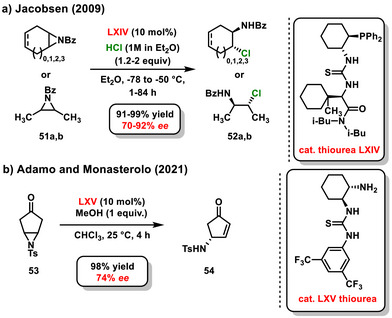
Enantioselective desymmetrization of *meso*‐aziridines catalyzed by chiral bifunctional organocatalysts.

Enantioselective desymmetrization can be applied not only to oxygen‐containing heterocycles but also to nitrogen analogs such as *meso*‐aziridines. In 2008, Wang and Wu published a study on a quinine‐catalyzed **II** asymmetric desymmetrization of *meso*‐aziridines **Xa,b** using various benzothiols. Their research also examined the effects of *meso*‐aziridines with differently substituted nitrogen atom.^[^ ^78^ ^]^ They successfully prepared the target β‐amino sulfides at good yields, ranging from 30% to 85%, with enantiomeric excesses between 19% and 72% ee. However, when using the Boc protecting group on the aziridine structure, the desymmetrization reaction exhibited no enantioselectivity. A year later, Jacobsen introduced an enantioselective method for opening the *meso*‐aziridine ring using hydrogen chloride. This desymmetrization reaction was successfully catalyzed by a chiral bifunctional thiourea **LXIV** containing a phosphine functional group. As a result, β‐chloroamine derivatives **52a,b** were obtained in high yields (91–99%) and with high enantiomeric purity (70–92% *ee*). The authors also found that a relatively high dilution (0.0025 M) resulted in the increased enantiocontrol of the reaction (Scheme 19a).^[^ ^79^ ^]^ Another intriguing study focused on the organocatalytic enantioselective desymmetrization of *meso*‐aziridines **53**, based on elimination reaction, was reported by Adamo and Monasterolo in 2021. The asymmetric intramolecular rearrangement catalyzed by bifunctional chiral thiourea **LXV** effectively generated chiral substituted 4‐aminocyclopentenones **54** with a yield of 98% and an enantiomeric excess of 74% *ee* (Scheme 19b).^[^ ^80^ ^]^ The introduction of an equivalent of methanol significantly accelerates the reaction. The authors proposed a mechanism involving covalent amine catalysis, attributed to the structure of the catalyst, which contains a primary amine. Additionally, they suggested a noncovalent bifunctional activation mechanism using chiral thiourea, highlighting that the presence of methanol contributes to the acceleration of the reaction.

In summary, the enantioselective desymmetrization of small heteroatom‐containing rings including epoxides, oxetanes, aziridines, and azetidines, the main challenge arises from their inherent ring strain and high reactivity. While ring strain facilitates ring opening or functionalization, excessive reactivity often compromises selectivity or promotes side reactions. As with anhydride substrates, the requirement for C_2_ or *meso* symmetry in the small‐ring starting materials remains a fundamental limitation.

## 4 Desymmetrization of Diols and Diamines

**Scheme 20:**
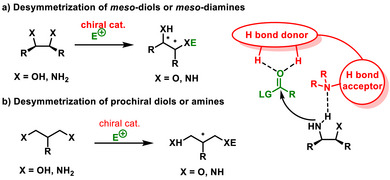
General scheme of desymmetrization of diols and diamines.

Diamines can also be used as valuable substrates in desymmetrizations (Scheme 20). Recently, Yang summarized in his review article the development of catalytic kinetic resolutions and desymmetrizations of amines.^[^ ^81^ ^]^

**Scheme 21:**
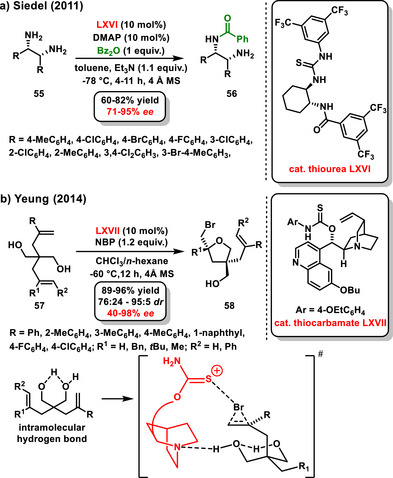
a) Enantioselective desymmetrization of *meso*‐diamines catalyzed by chiral thiourea and DMAP. b) Enantioselective desymmetrization of diolefinic diols catalyzed by chiral thiocarbamate.

Interestingly, in the area of H‐bonding catalysis, there is only work focused on the desymmetrization of *meso*‐diamines **55** with anhydrides developed by Siedel in 2011. The study involves the monobenzoylation of the amine functional group, facilitated by the cooperation of chiral thiourea **LXVI** and DMAP as organocatalysts. Although it is not a bifunctional activation of the substrates, activation via hydrogen bonding is involved. Desymmetrized monobenzoylated chiral diamines **56** were obtained in good yields (60–82%) and enantioselectivity (71–95% *ee*) (Scheme 21a).^[^ ^82^ ^]^

Enantioselective desymmetrization of *meso*‐diols was reviewed by Villegas,^[^ ^17^ ^]^ Xu with Lu^[^ ^83^ ^]^ and Suzuki.^[^ ^84^ ^]^ Desymmetrization of *meso*‐diols mainly focuses on acetylation or silylation of 1,2‐, 1,3‐diols, and polyols. While the vast majority of work in this area deals with bifunctional catalysis, activation of one of the substrates often occurs via the formation of a covalent bond rather than just a hydrogen bond. In 2014, Yeung reported work focused on the enantioselective desymmetrization of diolefinic diols **57** using thiocarbamate catalyst **LXVII** derived from cinchona alkaloids. The developed process involved the intramolecular bromoetherification of olefinic diols **57** with *N*‐bromophthalimide to produce substituted tetrahydrofuran derivatives **58**. The target tetrahydrofurans **58** were obtained at good yields and with good diastereomeric and enantiomeric ratios. Additionally, the products were subjected to cyclization to obtain hetero‐spirocyclic compounds (Scheme 21b).^[^ ^85^ ^]^ The proposed mechanism, supported by control experiments, assumes a significant role of intramolecular hydrogen bonding. This allows the starting material to adopt a pseudo‐chair conformation with a large olefinic side chain in a pseudo‐equatorial position. The second hydroxy group is activated by a tertiary amine of the quinuclidine scaffold. The bromonium ion can be stabilized by interaction with the amino thiocarbamate functional group.

## 5 Desymmetrization of Carbonyl Compounds

**Scheme 22:**
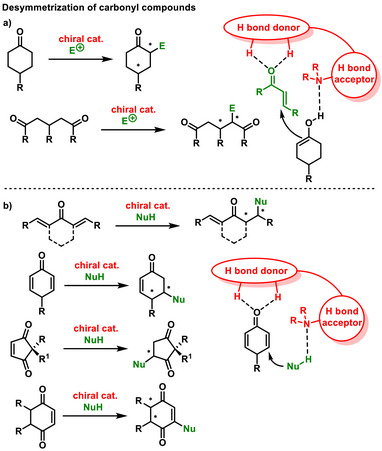
General scheme of desymmetrization of carbonyl compounds.

Bifunctional hydrogen catalysis has also been successfully used in desymmetrization reactions of carbonyl compounds. Summarizing works including also this subject has been partially described in review articles by Wang and Li,^[^ ^86^ ^]^ Dixon,^[^ ^3^ ^]^ Nájera,^[^ ^87^ ^]^ Xu and Ye^[^ ^88^ ^]^ and also Coeffard.^[^ ^89^ ^]^ To date, enantioselective desymmetrization of carbonyl compounds has been applied to a number of substrates and has yielded a range of structurally interesting chiral substances (Scheme 22). The desymmetrization reactions of carbonyl compounds can be classified according to whether the desymmetrized carbonyl substrate acts as an electrophile or a nucleophile in the reaction. In a situation where the substrate is an enolizable carbonyl compound (ketones and diketones), it is a nucleophilic substrate that is activated via the H‐acceptor group of the organocatalyst (Scheme 22a). Activation by the H‐donor group of the organocatalyst occurs in substrates such as dienones, bis‐enones, cyclohexadienones, cyclopentendiones, and cyclohexendiones. In this case, the carbonyl substrates act as electrophiles and are subject to nucleophile attack (Scheme 22b).

### 5.1 Ketones and Diketones

**Scheme 23:**
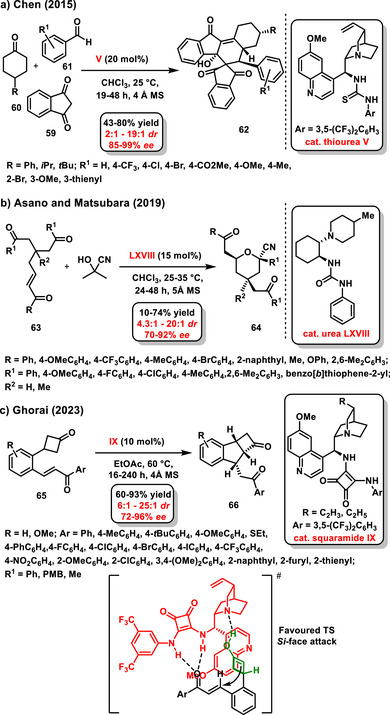
a), b) c) Enantioselective desymmetrization of ketones or diketones catalyzed by chiral bifunctional organocatalysts.

An exciting application of enantioselective desymmetrization of cyclohexanone is the enantioselective preparation of functionalized spiropolycarbocycles published by Chen's group in 2015. This three‐component organocatalytic reaction of 1,3‐indanedione **59**, cyclohexanone **60**, and an aromatic aldehyde **61** involves a Knoevenagel/Michal/double aldol reaction sequence and is catalyzed by a chiral bifunctional thiourea derived from *epi*‐quinine **V**. The target spiropolycyclic compounds **62** were prepared in high yields (43–80%) and with high stereoselectivity (2:1–19:1 *dr*, 85–99% *ee*) (Scheme 23a).^[^ ^90^ ^]^

In 2019, authors Asano and Matsubara reported a study on the enantioselective desymmetrization of achiral 1,5‐diketones **63**, affording functionalized tetrahydropyrans **64** containing three stereocenters. The developed organocatalytic cycloetherification was catalyzed by a chiral bifunctional urea **LXVIII** and involved the in situ generation of a cyanohydrin as an intermediate. The resulting tetrahydropyrans **64** were produced in good yields (10–74%) with high stereoselectivity (70–92% *ee*, 4.3:1 – 20:1 *dr*) (Scheme 23b).^[^ ^91^ ^]^ Cyclobutanones may also undergo enantioselective desymmetrization reaction. In 2023, Ghorai published an organocatalytic desymmetrization of 3‐substituted cyclobutanone derivatives **65**, followed by intramolecular cyclization catalyzed by a chiral bifunctional squaramide derived from *epi*‐quinine **IX**. The target tricyclic compounds **66** containing novel five‐ and four‐membered rings were obtained in good yields (60–93%) and with high stereoselectivity (6:1 – 25:1 *dr*; 72–96% *ee*) (Scheme 23c). Additionally, 3,3‐disubstituted cyclobutanones have also been successfully tested to achieve tricyclic products bearing all‐carbon quaternary centers.^[^ ^89^ ^]^

**Scheme 24:**
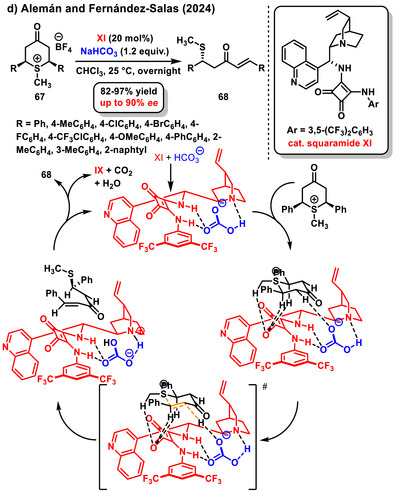
Enantioselective desymmetrization of cyclic keto sulfonium salts catalyzed by a combination of chiral squaramide and sodium bicarbonate.

Recently, a very interesting work was reported by Alemán and Fernández‐Salasby, the enantioselective desymmetrization of cyclic keto sulfonium salts **67**. The asymmetric desymmetrization reaction is associated with ring opening to form β‐methylsulfenylated enones **68**. A squaramide derived from *epi*‐cinchonine in combination with bicarbonate anion was used as a chiral bifunctional organocatalyst **XI**. The bicarbonate anion is activated by the chiral squaramide organocatalyst molecule, and deprotonation of hydrogen at the *α*‐position of the cyclic keto sulfonium salt occurs via the oxygen anion of bicarbonate. The target products were obtained at good yields (82–97%) and with high enantiomeric ratios (up to 90% *ee*) (Scheme 24).^[^ ^4^ ^]^ The authors conducted an in‐depth mechanistic study of the desymmetrization reaction and proposed its detailed pathway. According to their model, the carbonate anion is coordinated with the organocatalyst structure through hydrogen bonding, as confirmed by NMR experiments. The starting material is then bound in its cationic form and further stabilized in the transition state by hydrogen bonds. In the transition state, deprotonation of the α‐proton occurs with the simultaneous formation of a multiple bond and opening of the sulfonic cation ring. The catalytic cycle is completed by the regeneration of the catalyst and the release of CO_2_ and water molecules.

Another suitable substrate for asymmetric desymmetrization reactions is also 1,3‐diketone. In 2020, Asano and Matsubara developed an enantioselective desymmetrization of substituted cyclic 1,3‐diketones using trimethylsilyl cyanide through cyanohydrin as an intermediate. The reaction provided chiral oxadecalin derivatives at high yields (46–94%) and enantiomeric excess up to 98% using a chiral bifunctional thiourea as a catalyst.^[^ ^92^ ^]^ Simultaneously, Kowalczyk published the synthesis of chiral 1,4‐dihydropyridines via enantioselective desymmetrization of prochiral cyclic 1,3‐diketones. The organocatalytic Michael addition to unsaturated 2‐oxoesters was catalyzed by a chiral squaramide derived from cinchona alkaloids and afforded the target 1,4‐dihydropyridines at good yields and selectivity.^[^ ^93^ ^]^

### 5.2 Dienones and Bis‐enones

**Scheme 25:**
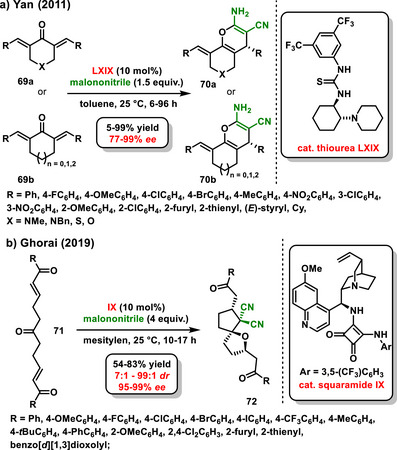
Enantioselective desymmetrization of cyclic or acyclic dienone catalyzed by chiral bifunctional organocatalysts.

In 2011, Yan utilized conformationally constrained heterocyclic dienones **69a,b** in an enantioselective desymmetrization reaction with malononitrile via a sequence of conjugated addition and intramolecular cyclization. The developed process was catalyzed by a bifunctional chiral thiourea **LXIX**, affording chiral pyran derivatives **70a,b** in high yields (5–98%) and enantioselectivities (77–99% *ee*) (Scheme 25a).^[^ ^94^ ^]^ Two years later, Yan extended this work using quinine **II** to catalyze the desymmetrization reaction, producing opposite enantiomers ***ent*‐70**.^[^ ^95^ ^]^ Conceptually similar work to Yan was reported by Du in 2014, who developed an enantioselective desymmetrization of similar cyclic dienones with malononitrile but catalyzed by a chiral bifunctional squaramide. The advantage of using such a catalyst was high efficiency even in a very small amount (1 mol%). The corresponding bicyclic pyran derivatives were synthesized at good yields (68–99%) with good enantioselectivity (23–98% *ee*).^[^ ^96^ ^]^

Another inspiring example of organocatalytic desymmetrization is the enantioselective desymmetrization of keto‐bisenones **71** with malonitrile described by Ghorai in 2019. An asymmetric cascade reaction involving a 1,4‐conjugated addition and a 1,2‐addition, followed by an *oxa*‐Michael addition, was successfully catalyzed by a chiral squaramide organocatalyst derived from *epi*‐quinine **IX**. *oxa*‐Spirocyclic products **72** bearing four‐carbon stereogenic centers were prepared in good yields (41–97%) with high diastereomeric ratios (7:1 – 99:1 *dr*) and enantiomeric purity (85–99% *ee*) (Scheme 25b).^[^ ^97^ ^]^ Another work, reported by Namboothiri, discusses the organocatalytic desymmetrization of curcumins with 3‐alkylidene oxindoles. The enantioselective transformation involves a double Michael addition cascade providing derivatives of spirocyclohexanone oxindoles. A chiral bifunctional thiourea was found to be a suitable catalyst with respect to the yield and selectivity of the reaction. A wide variety of differently functionalized spirocyclic oxindoles were prepared in good yields (60–90%) with high stereoselectivity (single diastereoisomers; 79–99% *ee*).^[^ ^98^ ^]^ Noteworthy, Namboothiri published studies focused on the enantioselective desymmetrization of curcumins through double Michael addition using substituted nitrostyrenes^[^ ^99^ ^]^ and 2‐alkylidene 1,3‐indanones^[^ ^100^ ^]^ previously.^[^ ^97^,^98^ ^]^ Unfortunately, the enantioselectivity obtained under catalysis with thioureas derived from cinchona alkaloids and quinine was only moderate.

### 5.3 Cyclohexadienones

**Scheme 26:**
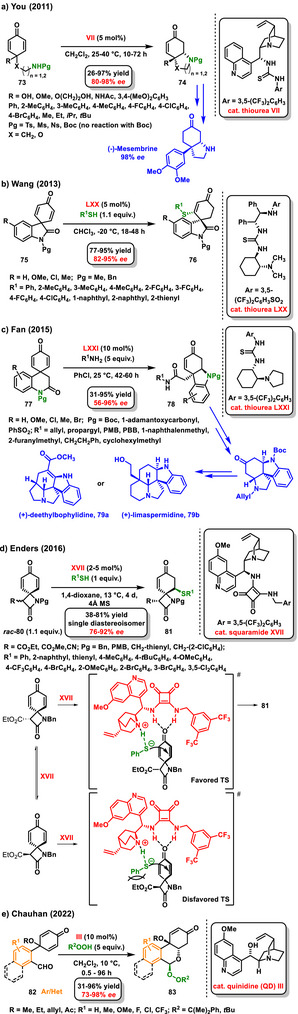
Enantioselective desymmetrization of derivatives cyclohexadienones catalyzed by chiral bifunctional organocatalyst.

**Scheme 27:**
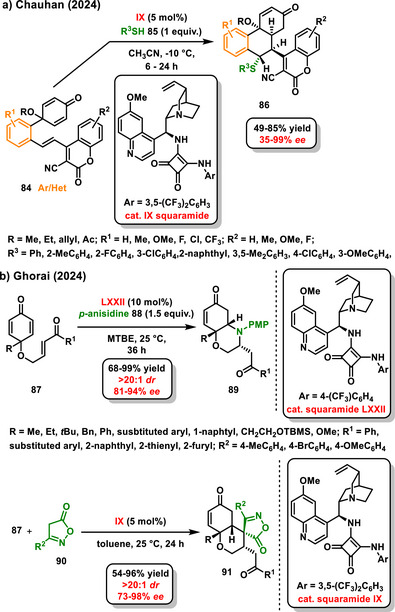
Enantioselective desymmetrization of derivatives of cyclohexa‐2,5‐dienones catalyzed by chiral bifunctional squaramides.

In 2011, You utilized cyclohexadienones for enantioselective desymmetrizations. He proposed an intramolecular enantioselective desymmetrization method using the *aza*‐Michael reaction on cyclohexadienones **73**, which was catalyzed by a bifunctional chiral thiourea derived from cinchonine **VII** (Scheme 26a).^[^ ^101^ ^]^ This approach provided a small library of bicyclic pyrrolidine and morpholine derivatives **74** in high yields (26–97%) with high enantiomeric purity (80–98% *ee*). Additionally, this method was successfully applied in the total synthesis of (‐)‐mesembrine (98% *ee*). The work was expanded by the same authors through the investigation of the intramolecular asymmetric desymmetrization of cyclohexadienones via the Michael addition of carbon‐based Michael donors. Similarly, highly enantiomerically enriched polycyclic cyclohexenones were obtained in high yields (82–97%).^[^ ^102^ ^]^ Two years later, Wang used spirocyclohexadienone oxindoles **75** in an enantioselective desymmetrization process via a sulfa‐Michael addition. A thiourea derivative **LXX** emerged as an effective chiral bifunctional catalyst, producing chiral spiro compounds **76** in good yields (77–95%) with high enantiomeric purity (82–95% *ee*) (Scheme 26b).^[^ ^103^ ^]^ A practical example of asymmetric desymmetrization is evident in the total synthesis of (±)‐morphine, published by Fan in 2013. In this study, the enantioselective desymmetrization of a spirocyclohexadienone derivative was employed to construct ring E of the morphine structure. A chiral bifunctional thiourea derived from *epi*‐quinine **V** effectively catalyzed the tandem alcoholysis/oxa‐Michael addition reaction. The key polycyclic intermediate was obtained at good yield (90%) with an enantiomeric excess of 48% *ee*.^[^ ^104^ ^]^ In 2015, Fan built upon his previous work and developed the total synthesis of hydrocarbazole alkaloids **79a,b** from the *Apocynaceae* family. In this case, he utilized enantioselective organocatalytic desymmetrization of prochiral spirocyclic dienonimides **77** through a tandem reaction that combined aminolysis and *aza*‐Michael addition catalyzed by bifunctional thiourea **LXXI**. The resulting functionalized hydrocarbazoles **78** were produced in good yields and with high enantiomeric excess (Scheme 26c).^[^ ^105^ ^]^ Also, Enders investigated enantioselective desymmetrizations, specifically desymmetrization of spirocyclohexadienone β‐lactam substrates **80** via sulfa‐Michael addition process. The stereoselective reaction was catalyzed by a chiral bifunctional Rawal squaramide catalyst **XVII**, and the reaction afforded spirocyclohexenone β‐lactam derivatives **81** with three stereogenic centers in good yields (38–81%) and enantioselectivity (76–92% *ee*) (Scheme 26d).^[^ ^106^ ^]^ In 2023, the *sulfa*‐Michael addition was also used for the asymmetric desymmetrization of spirocyclic cyclohexadienone isobenzofuranones with aromatic thiols, as reported by Chauhan. A chiral bifunctional squaramide **IX** derived from cinchona alkaloids proved to be a suitable catalyst with respect to the yield and selectivity of the reaction.^[^ ^107^ ^]^ Other studies focusing on the intramolecular desymmetrization of cyclohexa‐2,5‐dienone were published by Chauhan. In 2022, he developed a quinidine‐catalyzed enantioselective reaction sequence involving peroxyhemiacetalization by peroxyalcohol followed by intramolecular *oxa*‐Michael addition to 4‐substituted cyclohexa‐2,5‐dienones **82**. The reaction provided highly functionalized isochroman derivatives **83** as a single diastereoisomer in good yields (31–96%) with high enantiomeric excess (73–98% *ee*) (Scheme 26e).^[^ ^108^ ^]^ The following work from the same group focused on the asymmetric synthesis of hydrophenanthrone derivatives, which contain five stereogenic centers. Again, a suitably substituted cyclohexa‐2,5‐dienones were used as starting substrates, which underwent enantioselective intermolecular desymmetrization via a 1,4‐/1,4 addition reaction sequence. The enantioselective reaction was successfully catalyzed by a derivative of the chiral bifunctional Rawal squaramide **XIX**. The corresponding hydrophenanthrone derivatives were prepared in good yields (28–98%) with high enantiomeric purity (1.4:1 – 20:1 *dr*; 72–99% *ee*).^[^ ^109^ ^]^ In 2024, Chauhan continued with the synthesis of hydrophenanthrone derivatives and proposed the enantioselective organocatalytic desymmetrization of 2,5‐cyclohexadienone tethered to 3‐cyano‐4‐styrylcoumarins **84** as a *sulfa*‐1,6‐addition sequence of thiols **85** to a 3‐cyano‐4‐styrylcoumarin moiety, followed by an intramolecular 1,4‐addition. The enantioselective reaction was catalyzed by a bifunctional squaramide **IX** and afforded the substituted hydrophenanthrenone derivatives **86** in good yields (49–85%) and high diastereoisomeric (>20:1 *dr*) and enantiomeric purity (35–99% *ee*) (Scheme 27a). Interestingly, when an excess of thiol was used, an additional *sulfa*‐1,4‐addition to the second multiple bond in a cyclohexa‐2,5‐diene structure occurred.^[^ ^110^ ^]^

Two works reported last year by Ghorai also dealt with the enantioselective desymmetrization of 4‐substituted derivatives of 2,5‐cyclohexadienones **87**. The first study focused on the organocatalytic desymmetrization through double *aza*‐Michael addition with anisidine **88** leading to morpholine derivatives **89**. The reaction was catalyzed by a chiral bifunctional squaramide derivative derived from *epi*‐quinine **LXXII** and afforded highly functionalized bicyclic morpholine derivatives **89** in good yields with good enantiomeric excess (Scheme 27b).^[^ ^111^ ^]^ In the second work, Ghorai studied enantioselective desymmetric spirocyclization on 4‐substituted 2,5‐cyclohexadienones bearing enone moiety **87** with isoxazolines **90**. Chiral squaramide **IX** derived from cinchona alkaloids proved to be more suitable with respect to the yield and the studied selectivity of the reaction. The target spiro compounds **91** contained four novel stereogenic centers and were obtained at good yields with high stereoselectivity (20:1 *dr*, up to 98% ee, Scheme 27b).^[^ ^112^ ^]^

**Scheme 28:**
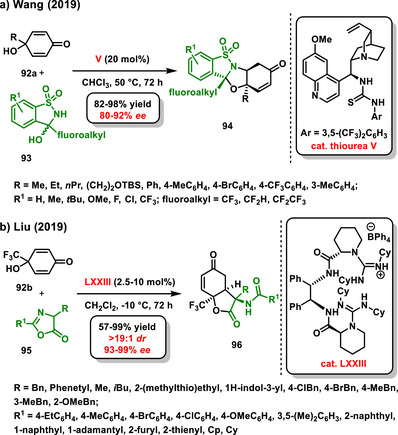
Enantioselective desymmetrization of derivatives *p*‐quinols catalyzed by chiral bifunctional organocatalyst.

In 2019, Wang reported desymmetrization of *p*‐quinol derivatives **92a** using fluoroalkylated *N,O*‐ketals **93** based on saccharine scaffold. The process involved dehydration/aminalization/*aza*‐Michael addition reaction sequence efficiently catalyzed by bifunctional thiourea derived from *epi*‐quinine **V**. The resulting fluoroalkylated heterocyclic hemiaminals **94** were obtained in high yields with good enantioselectivity (Scheme 28a).^[^ ^113^ ^]^ Another example of organocatalytic asymmetric desymmetrization of *p*‐quinols **92b** by azlactones **95** was described by Liu. The developed process involving enantioselective cascade Michael addition followed by lactonization was efficiently catalyzed by bifunctional chiral bis‐guanidine hemisalt **LXXIII**. The corresponding products **96** were isolated in good yields (57–99%) with high diastereo‐ (>19:1 *dr*) and enantioselectivity (93–99% *ee*) (Scheme 28b).^[^ ^114^ ^]^ A few years later, Liu and Chen studied DABCO‐catalyzed [3 + 2] cycloaddition/deformylation cascades, and as a part of it they described a single example of enantioselective desymmetrization of *p*‐quinol derivatives with 3‐formylchromones. The enantioselective reaction was catalyzed by the chiral bifunctional Takemoto thiourea **XLIII** and afforded the desired benzopyrone‐fused hydrobenzofuranone at moderate (53%) yield with promising enantioselectivity (81% *ee*).^[^ ^115^ ^]^

### 5.4 Cyclopentendiones

**Scheme 29:**
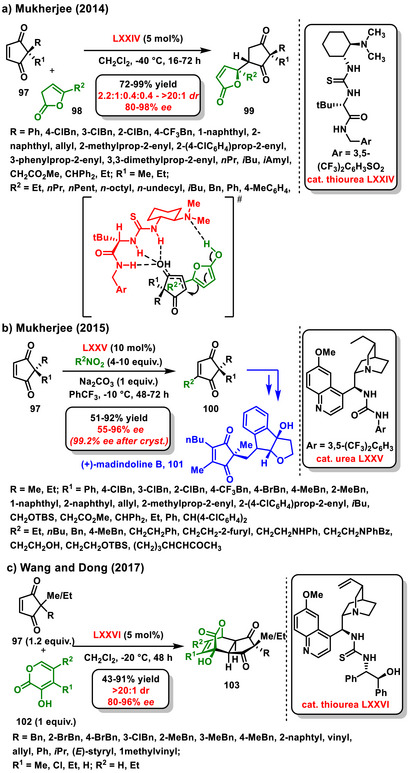
Enantioselective desymmetrization of 2,2‐disubstituted cyclopentene‐1,3‐diones catalyzed by chiral bifunctional organocatalyst.

Cyclopentenediones are also effective molecules for enantioselective desymmetrization, with the Michael reaction being the most common method used to achieve that. The pioneering study in this area, developed by Mukherjee, is focused on the enantioselective desymmetrization of 2,2‐disubstituted cyclopenten‐1,3‐diones **97**, utilizing α‐angelica lactone **98** as a nucleophile in a conjugate addition. In this study, chiral bifunctional thiourea **LXXIV** was chosen as a suitable catalyst due to its positive impact on both the yield and selectivity of the reaction. Notably, the N‐H hydrogen in the amide structure of the catalyst plays an important role in activating the cyclopentenedione, leading to increased selectivity in the enantioselective reaction. Conversely, when the catalyst had an *N*‐methylated amide structure, there was a significant decrease in enantioselectivity, and diastereoselectivity was also slightly reduced. The authors further investigated the robustness of the enantioselective desymmetrization in the presence of additional Michael acceptors and donors. The desired substituted cyclopentanediones were consistently produced at good yields (72–99%) with high diastereo‐ (up to > 20:1 *dr*) and enantioselectivity (80–98% *ee*) (Scheme 29a).^[^ ^116^ ^]^

A year later, Mukherjee performed enantioselective desymmetrization alkylation of 2,2‐disubstituted cyclopenten‐1,3‐diones **97** with nitroalkanes as alkylating agents. The chiral bifunctional urea **LXXV** successfully catalyzed this reaction, which provided cyclopentene rings bearing one stereogenic center at good yields (51–92%) with moderate to high enantioselectivity (55–96% *ee*). Additionally, synthetic utility was demonstrated by the preparation of (+)‐mandindoline B **101**, which contains a dialkylated cyclopentene‐1,3‐dione skeleton (Scheme 29b).^[^ ^117^ ^]^ In 2017, Enders followed up on Mukherjee's work and developed enantioselective desymmetrization of cyclopent‐4‐ene‐1,3‐diones via Michael addition of oxindoles and pyrazolones.^[^ ^118^ ^]^ Whereas in the enantioselective desymmetrization with oxindoles catalysis by a chiral bifunctional squaramide **IX** was used, desymmetrization with pyrazolones was promoted by bifunctional thiourea derived from *epi*‐quinine **V**. In both cases, the corresponding products were obtained in good yields (14–95%) with high diastereoselectivity (8:1 – >20:1 *dr*) and enantioselectivity (50–94% *ee*).^[^ ^116^,^117^ ^]^ Authors Dong and Wang published another elegant example of enantioselective desymmetrization of prochiral 2,2‐disubstituted cyclopenten‐1,3‐diones **97**. The developed process proceeded through the Diels‐Alder reaction with 3‐hydroxy‐2‐pyrones **102** catalyzed by chiral bifunctional thiourea derived from cinchona alkaloid **LXXVI**. The reaction afforded highly functionalized bridged tricyclic compounds **103** containing five stereogenic centers with high selectivity (Scheme 29c).^[^ ^119^ ^]^

**Scheme 30:**
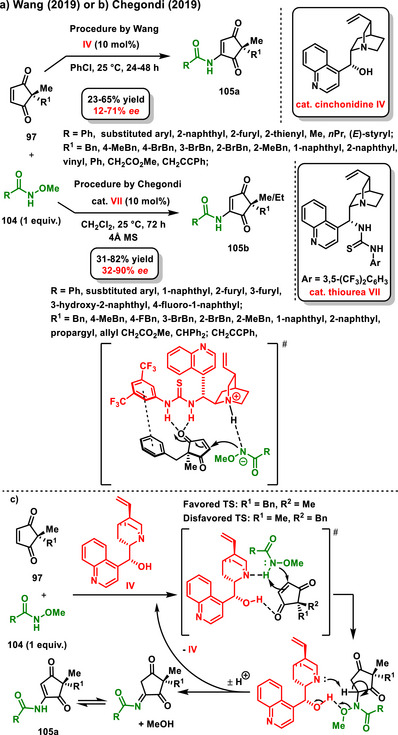
a) and b) Enantioselective desymmetrization of 2,2‐disubstituted cyclopentene‐1,3‐diones with *N*‐methoxy amides catalyzed by chiral bifunctional organocatalyst. c) Proposed mechanism of C(sp^2^)‐H amidation catalyzed by cinchonine.^[^ ^122^ ^]^

In 2019, Wang and Chegondi independently described enantioselective desymmetrization of 2,2‐substituted cyclopenten‐1,3‐dione **97** through *N*‐amidation with *N*‐methoxybenzamides **104**. Cinchonidine **IV** was used as a suitable organocatalyst in the work of Wang, whereas Soós chiral bifunctional thiourea **VII** promoted the process described by Chegondi. Generally, highly functionalized five‐membered carbocycles **105a,b** were prepared at good yields and with medium to high enantiomeric ratios. (Scheme 30a,b).^[^ ^120^, ^121^ ^]^ Wang also proposed a plausible mechanism for the desymmetrization reaction, supported by NMR experiments and previous studies. In this mechanism, both starting materials interact with the catalyst through hydrogen bonding to form the transition state. This is followed by an enantioselective Michel addition of *N*‐methoxy amide to cyclopentenedione, forming an intermediate. Subsequent catalyst‐assisted elimination of the methanol affords an imine derivative that undergoes tautomerization to give the final product (Scheme 30c).

**Scheme 31:**
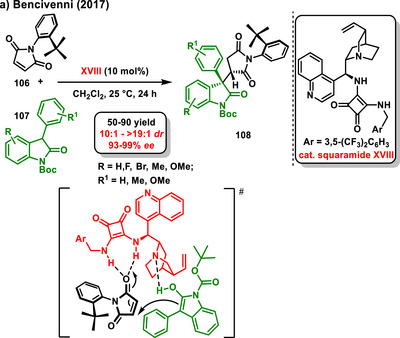
Enantioselective desymmetrization of substituted maleinimides with oxindoles catalyzed by chiral bifunctional squaramide.

In addition to cyclopentene‐1,3‐dione, as a molecule where enantioselective desymmetrization can be easily performed, a suitable maleinimide derivative can also be used. In 2017, Bencivenni published Michael addition of oxindole derivatives **107** to *N*‐(2‐*tert*‐butylphenyl)maleimides **106**, which led to the preparation of the corresponding desymmetrized axially chiral succinimides in good yields. The reaction was successfully catalyzed by the chiral bifunctional Rawal squaramide **XVIII**, affording the products with high diastereo‐ and enantiocontrol (Scheme 31).^[^ ^122^ ^]^ The proposed transition state corresponds to the attack of the enolate on the β‐carbon of the *Re*‐face of the maleinimide.

### 5.5 Cyclohexendiones

**Scheme 32:**
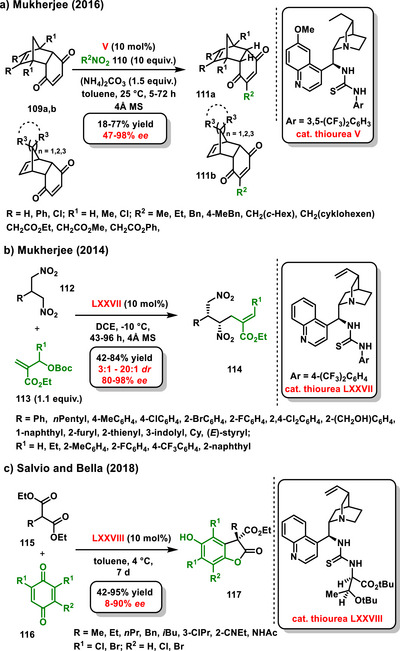
a) Enantioselective desymmetrization of *meso*‐bicyclic cyklohex‐2‐en‐1,4‐diones with nitroalkanes catalyzed by a chiral bifunctional organocatalyst. b) and c) Enantioselective desymmetrization of 1,3‐disubstituted compounds catalyzed by a chiral bifunctional organocatalyst.

Cyclohex‐2‐ene‐1,4‐diones can also be used for enantioselective desymmetrization proceeding through Michael addition. This structural motif can be found in Diels‐Alder cycloaddition products, for example, *meso*‐norbornenoquinones **109**. In 2016, Mukherjee performed enantioselective desymmetrization of *meso*‐norbornenoquinones **109** via an alkylation reaction with nitroalkanes **110** catalyzed by chiral bifunctional thiourea **V** derived from cinchona alkaloids. This concept has already been used by him for the asymmetric desymmetrization of cyclopenten‐1,3‐dione derivatives. Alkylated products **111a,b** were obtained at good yields and with high enantiomeric ratios (Scheme 32a).^[^ ^123^ ^]^ Six years later, Mukherjee extended this methodology to *meso*‐cyclopropanes fused with cyclohexene‐1,4‐diones. He subjected these substrates to desymmetrization by alkylation with nitroalkanes. However, the enantioselective desymmetrization alkylation was catalyzed by a bifunctional squaramide (*epi*‐quinine), affording alkylated products with high selectivity and yield.^[^ ^124^ ^]^

### 5.6 Malonates and 1,3‐Dinitro Compounds

**Scheme 33:**
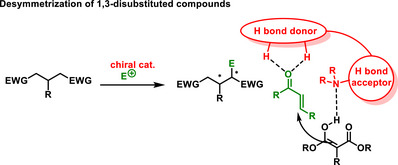
General scheme of desymmetrization of 1,3‐disubstituted compounds.

Other suitable substrates for enantioselective desymmetrization may be 1,3‐disubstituted compounds, such as 1,3‐dinitro compounds or malonates (Scheme 33). In 2014, Mukherjee developed an organocatalytic enantioselective desymmetrization reaction of substituted 1,3‐dinitropropanes **112** using Morita‐Baylis‐Hillman allyl carbonates **113**. The desymmetrization based on allylic alkylation reaction was catalyzed by chiral bifunctional thiourea derived from *epi*‐quinine **LXXVII**. The corresponding products **114** with two vicinal stereogenic centers were isolated in moderate yields (42–84%) and with high diastereoselectivity (3:1 – >20:1 *dr*) and enantioselectivity (80–98% *ee*) (Scheme 32b).^[^ ^125^ ^]^ In fact, MBH carbonate is likely activated in the reaction through a covalent bond with a tertiary amine in the structure of the organic catalyst, but the hydrogen atoms of the thiourea unit can activate the nitro group via hydrogen bonds.

A few years later, enantioselective desymmetrization of 2‐substituted malonates **115** was reported by Salvio. In their study, malonates were desymmetrized using 1,4‐benzoquinones **116** through an organocatalytic Michael addition, leading to the formation of benzofuranone derivatives **117**. This reaction was catalyzed by a chiral bifunctional thiourea derived from cinchona alkaloids **LXXVIII**. An interesting aspect of this enantioselective reaction is that the method of adding the benzoquinone has a significant influence on the yield. When benzoquinone is added in a single portion, very low yields (less than 20%) of benzofuranone derivatives were obtained. In contrast, when 1,4‐benzoquinone is added in portions, the yield of benzofuranone increased dramatically, reaching up to 95% (Scheme 32c).^[^ ^126^ ^]^

## 6 Summary and Outlook

Enantioselective organocatalytic desymmetrization is an efficient tool for forming chiral entities from achiral substrates with high efficiency and selectivity. Chiral bifunctional organocatalysts based on hydrogen bonding, especially those combining donor and acceptor functional groups (e.g., bifunctional thioureas, squaramides, and amides), have proven to be extremely effective in controlling the stereoselectivity of these reactions. Their ability to simultaneously activate both nucleophiles and electrophilic substrates via hydrogen bonding is key to achieving high enantioselectivity.

Despite considerable progress in recent years, there remains room for further development. This includes, in particular, the expansion of the substrate spectrum of molecules that can undergo desymmetrization reactions. Furthermore, it is worthwhile to focus on the discovery of new catalytic systems, such as the coupling of organocatalysis and transition metal catalysis, as well as other innovative catalytic concepts. Overall, developments in this area can be expected to continue contributing to the more efficient synthesis of chiral compounds in modern organic chemistry, with overlap into total synthesis of natural and structurally interesting molecules.

## Conflict of Interest

The authors declare no conflict of interest.

## Data Availability

Data sharing is not applicable to this article as no new data were created or analyzed in this study.
